# Influence of W Addition on Phase Constitution, Microstructure and Magnetic Properties of the Nanocrystalline Pr_9_Fe_65_W_x_B_26-x_ (Where: x = 2, 4, 6, 8) Alloy Ribbons

**DOI:** 10.3390/ma13102229

**Published:** 2020-05-13

**Authors:** Katarzyna Filipecka, Piotr Pawlik, Andrzej Kozdraś, Waldemar Kaszuwara, Jarosław Ferenc, Jacek Filipecki

**Affiliations:** 1 Faculty of Mathematics and Natural Science, Jan Dlugosz University, Armii Krajowej 13/15, 42-200 Czestochowa, Poland; katarzyna.filipecka@onet.eu; 2 Faculty of Production Engineering and Materials Technology, Czestochowa University of Technology, Armii Krajowej 19, 42-200 Czestochowa, Poland; piotr.pawlik@pcz.pl; 3 Faculty of Production Engineering and Logistics, Opole University of Technology, Ozimska 75, 45-370 Opole, Poland; a.kozdras@po.opole.pl; 4 Faculty of Materials Science and Engineering, Warsaw University of Technology, Woloska 141, 02-507 Warsaw, Poland; waldemar.kaszuwara@pw.edu.pl (W.K.); jaroslaw.ferenc@pw.edu.pl (J.F.)

**Keywords:** rare earth, permanent magnets, rapid solidification, magnetic properties, X-ray diffraction, Mössbauer spectroscopy, crystallization, thermal stability, transmission electron microscopy

## Abstract

The aim of the present work was to investigate an influence of W addition on the phase constitution, microstructure and magnetic properties of the Pr_9_Fe_65_W_x_B_26-x_ (where: x = 2, 4, 6, 8) alloy ribbons. Ribbons were obtained using the melt-spinning technique under low pressure of Ar. The as-cast samples were fully amorphous and revealed soft magnetic properties. These facts were confirmed by X-ray diffractometry, Mössbauer spectroscopy and magnetic measurements. Differential scanning calorimetry and differential thermal analysis allowed us to determine the thermal stability parameters of the amorphous phase. The Kissinger plots were constructed in order to calculate the activation energies for crystallization. Heat treatment carried out at various temperatures caused changes in the phase constitution and magnetic properties of the alloys. The phase analysis has shown the presence of the hard magnetic Pr_2_Fe_14_B and paramagnetic Pr_1+x_Fe_4_B_4_ phases. Additionally, for the x = 2 and x = 6 alloys, a crystallization of soft magnetic Fe_2_B and α-Fe phases was observed. The Mössbauer spectroscopy allowed us to determine the volume fractions of constituent phases formed during annealing. The microstructure of annealed ribbons was observed using transmission electron microscopy.

## 1. Introduction

For over half a century, much attention has been paid to the development of various types of high-performance permanent magnets based on rare-earth (RE) transition-metal (TM) intermetallic compounds. Their exceptional magnetic properties can be attributed to the favorable combination of properties such as high magnetocrystalline anisotropy, which is provided by the RE elements and high magnetic moments of TM elements also present in the unit cells of these compounds [1]. The most important advance in RE hard magnetic materials was made in 1984 when a new Nd_2_Fe_14_B ternary compound was announced by two independent groups—Sagawa et al. [2] at Sumitomo Special Metals Co., Ltd. (Osaka, Japan) and Croat et al. [3] at General Motors Co. (Warren, MI, USA). The first group mentioned above developed the anisotropic magnets based on aligned and sintered 10 µm diameter particles of the Nd_2_Fe_14_B phase. The magnetic parameters measured for this alloy were the coercivity _J_H_c_ = 1000 kA/m, the polarization remanence J_r_ = 1.2 T and the maximum magnetic energy product (BH)_max_ = 288 kJ/m^3^. The second group developed the melt-spun ribbons that contained ultrafine randomly oriented grains of the Nd_2_Fe_14_B phase of the average diameters less than 100 nm. The typical magnetic properties of these magnets were _J_H_c_ = 1120 kA/m, J_r_ = 0.8 T and (BH)_max_ = 122 kJ/m^3^. Further studies on the Nd-Fe-B type magnets were oriented towards improving their properties and finding more favorable manufacturing techniques. The composition of the alloy was modified many times over the years to give a whole family of RE-(Fe, TM)-B magnets [4,5,6]. In particular, Pr-Fe-B type magnets are of considerable interest, due to the fact that the Pr_2_Fe_14_B phase is responsible for the hard magnetic properties. The Pr_2_Fe_14_B phase has similar magnetic properties and the same crystalline structure as the Nd_2_Fe_14_B [7]. Furthermore, the saturation polarization J_s_ and Curie temperature T_C_ are also similar [8], but for the Pr_2_Fe_14_B phase, a higher magnetocrystalline anisotropy field was measured [9]. Furthermore, due to the fact that the Pr_2_Fe_14_B phase does not undergo a spin reorientation down to 25 K [10], the Pr-based alloys sustain good magnetic properties at low temperatures. Up to now, numerous pieces of work have been carried out in order to investigate the effect of micro-alloying on the microstructure and magnetic properties in the Pr-Fe-B magnets [11,12,13,14,15,16,17,18]. Some studies have been published on the influence of W addition on the microstructure and magnetic properties of the RE-Fe-B alloys [19,20,21,22,23,24,25,26,27]. It was shown in Reference [26] that small additions of W to the alloy composition can significantly improve the glass forming abilities of the alloy. Furthermore, its admixture to the Nd-Fe-B alloy resulted in some changes in the magnetic properties [27,28]. The studies carried out on Nd-Fe-B magnets produced by the mechanical alloying have revealed that a gradual increase of W in the alloy composition causes an increase of the coercivity _J_H_c_ in a wide range of W contents. It was also proven that this effect is accompanied by a gradual decrease of the remanence J_r_ and maximum magnetic energy product (BH)_max_ [27,28]. It should be noticed that obtaining the optimal magnetic parameters depends also on the production technology. An adequate and cheap processing technique is an important factor in the final market price of the magnets. The application of the rapid solidification techniques combined with heat treatment is a response to these needs. It allows us to obtain appropriate nanocrystalline structure and hard magnetic properties at shorter processing time, which can significantly reduce the production costs. Moreover, these costs can be further cut down by lowering the RE content in the alloy composition. Previous studies on Pr-(Fe,Co)-B-type alloys with high boron contents and additions of Ti revealed a high coercivity in the as-cast state [29]. It was shown for the Fe_91-x_Zr_5_B_x_Nb_4_ (x = 0 to 36) alloys, that increase of B content above 20 at. %, result in significant improvement of the glass forming abilities (GFA). Good GFA was observed up to 30 at. % of B, while a further increase of B resulted in a sharp drop of GFA [30]. A beneficial impact of B content on the GFA was also utilized in the RE-Fe-B-type magnets. In the case of the Fe_67_Co_9.5_Nd_3_Dy_0.5_B_20_ alloy it was possible to cast up to 0.5 mm diameter rods [31,32], while for Fe_61_Co_13.5_Zr_1_Pr_4.5-x_Dy_x_B_20_ (x = 0, 1) alloys [33], fully amorphous 1 mm diameter rods and up to 3 mm outer diameter tubes were suction-cast. In general, the RE-Fe-B magnets can be divided into two groups: the Fe-reach [34] and B-reach alloys [35]. The main advantages of the B-reach magnets are the low contents of expensive RE elements, their low melting points and good GFA. Furthermore, the devitrification of the B-reach RE-Fe-B alloys results in the formation of nanocomposite magnets consisting of Fe_3_B, Nd_2_Fe_14_B, and α-Fe phases [36]. For these reasons, the B-reach magnets can be an interesting alternative to the commonly used Fe-reach RE-Fe-B magnets. It is well established that Nb addition to the amorphous alloys results in retarding the diffusion of iron atoms [37]. This prevents the fast growth of α-Fe during casting. Furthermore, it was shown, for the rapidly solidified (Fe_86-x_Nb_x_B_14_)_0.88_Tb_0.12_ (2 ≤ x ≤ 8) alloys [38], that addition of Nb and appropriate rapid solidification are responsible for the magnetic hardening effect. This is caused by the formation of dendrites of the Tb_2_Fe_14_B hard magnetic phase, with Nb-rich interdendritic regions. On the other hand, the addition of Nb resulted in good glass forming abilities. Subsequent annealing allowed us to tailor the magnetic properties of the bulk glassy samples [39].

In the present work, the influence of the W addition on phase constitution, microstructure and magnetic properties of the rapidly solidified Pr_9_Fe_65_W_x_B_26-x_ (where: x = 2, 4, 6, 8) alloy ribbons, was discussed. Our goal was to determine the influence of W addition on the glass forming abilities of the Pr_9_Fe_65_W_x_B_26-x_ (where: x = 2, 4, 6, 8) alloys. Furthermore, for the devitrified samples, the influence of W content on the phase constitution and magnetic properties will be investigated.

## 2. Materials and Methods

The Pr_9_Fe_65_W_x_B_26-x_ (where: x = 2, 4, 6, 8) alloys were synthesized by arc-melting from pure elements (Pr ingot of the purity 99.9%; Fe granular of the purity 99.98%; W foil of the purity 99.9% were purchased at Sigma-Aldrich (St. Louis, MO, USA)) with the addition of the Fe-B pre-alloy (of the purity 98%, bought at Alfa-Aesar) under the Ar atmosphere. The alloys were subsequently melt-spun under an Ar atmosphere to obtain ribbon samples. In this process, the molten alloys were injected on the surface of the copper roll by the overpressure of the argon. The linear velocity of the copper roll surface was 25 m/s. The thickness of the ribbon depends on the composition of the alloys. The average thicknesses were 15 μm, 20 μm, 18 μm and 20 μm, respectively for the Pr_9_Fe_65_W_2_B_24_, Pr_9_Fe_65_W_4_B_22_, Pr_9_Fe_65_W_6_B_20_ and Pr_9_Fe_65_W_8_B_18_ alloys. In order to obtain a nanocrystalline microstructure and induce some changes in the magnetic parameters, the samples were subjected to heat-treatment. For this purpose, the ribbons were sealed-off in quartz tubes under a low pressure of Ar (0.5 bar) and subjected to annealing for 5 min at temperatures ranging from 943 K to 1023 K and then rapidly quenched in icy water. These annealing conditions have been proven beneficial for this alloy [40]. Thermal stability parameters were determined using differential scanning calorimetry (DSC) (DSC 404, Netzsch, Selb, Germany) and differential thermal analysis (DTA) (LabSys DTA/DSC, Setaram, Caluire, France) techniques. To establish the activation energies for crystallization, the DSC curves were measured with different heating rates (1 K/min ≤ α ≤ 10 K/min) for each alloy composition. The phase constitution of specimens was studied using X-ray diffractometry (XRD). The XRD patterns were measured using Bruker D8 Advance diffractometer (Bruker AXS GmbH, Karlsruhe, Germany) with CuK_α_ radiation, LynxEye semiconductor detector, (linear focus of 25 mm, primary beam divergence slit of 0.6 mm) and Soller slits on the primary and diffracted beam. The measurements were performed in the Bragg-Brentano configuration with the Ni-K_β_ filter located on the detector side. A continuous scan mode in the 2θ range between 30 and 100 deg with the step size of 0.02 deg and a step time of 5 s were used. The phase analysis was performed using EVA software (Bruker AXS GmbH, Karlsruhe, Germany). The Mössbauer spectra were measured at room temperature using a Polon 2330 Mössbauer spectrometer (Polon, Poland) working in transmission geometry, utilizing the ^57^Co:Rh source. The Mössbauer spectra were fitted with Win Normos for Igor 3.0 package. In order to obtain a specimen representative for the entire volume of the material, the samples were crushed to powder. Measurements of magnetic hysteresis loops were performed using a LakeShore 7307 Vibrating Sample Magnetometer (VSM, Lakeshore Cryotronics, Westerville, OH, USA), operating in external magnetic fields up to 2 T at room temperature. The observation of the microstructure was carried out using transmission electron microscopy (TEM, JEOL JEM 1200 EX, JEOL Inc., Peabody, MA, USA). The diagrams of the crystallite size distributions were constructed base on the analysis of the TEM images, taken from different areas of the samples. To reduce the measurement uncertainties, a large series of crystallite size measurements were carried out (~750 for each sample).

## 3. Results and Discussion

**Figure 1:**
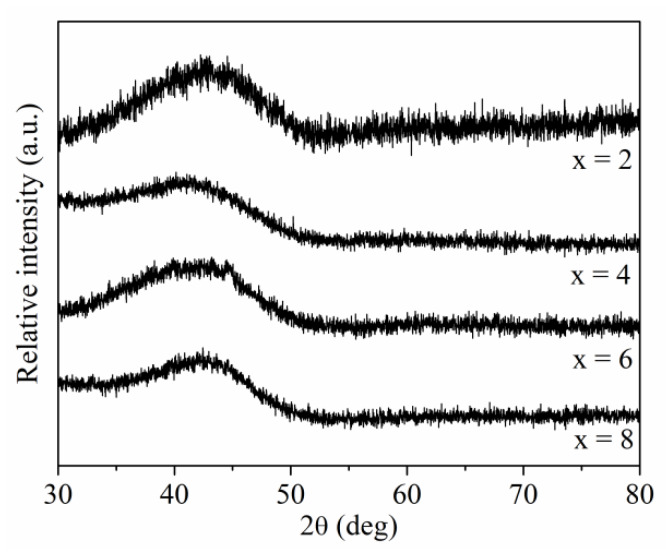
X-ray diffraction patterns measured for the Pr_9_Fe_65_W_x_B_26-x_ (where: x = 2, 4, 6, 8) ribbons in an as-cast state.

The XRD patterns measured for the rapidly solidified Pr_9_Fe_65_W_x_B_26-x_ (where: x = 2, 4, 6, 8) alloy ribbons in the as-cast state are shown in Figure 1. These samples had an amorphous structure, which was indicated by the presence of wide bumps on XRD scans in the range of 2θ between 35 and 50 deg, characteristic for the amorphous phase.

**Figure 2:**
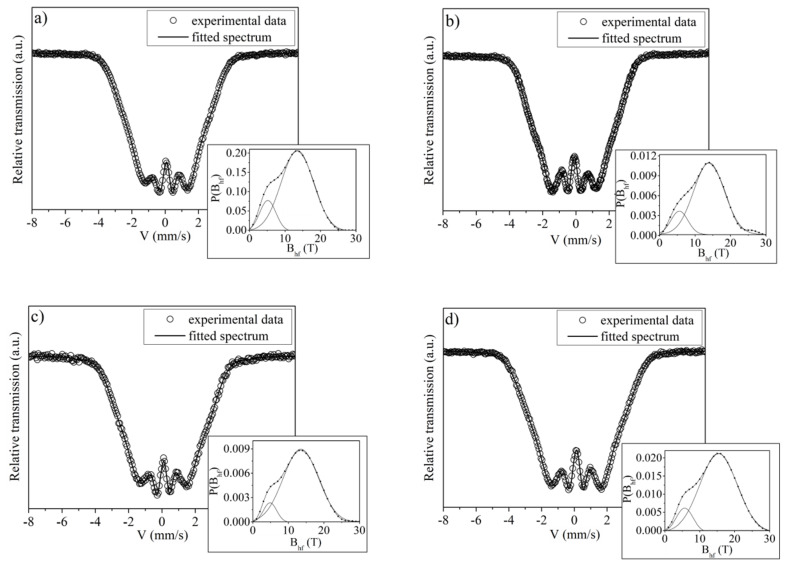
Mössbauer spectra with corresponding hyperfine field distributions of the Pr_9_Fe_65_W_x_B_26-x_ (where: x = 2 (**a**), x= 4 (**b**), x = 6 (**c**) and x = 8 (**d**)) ribbons in an as-cast state.

Analysis of the Mössbauer spectra measured for the as-cast Pr_9_Fe_65_W_x_B_26-x_ (where: x = 2, 4, 6, 8) (Figure 2) ribbons confirmed their amorphous structure. In all cases, large broadenings of the Mössbauer lines were measured. In the fitting procedure, the hyperfine field distributions were calculated and experimental spectra were fitted by broad lines corresponding to these distributions. The bimodal character of the distributions suggests the existence of nonequivalent surroundings of Fe atoms in the amorphous phase. Furthermore, there is no significant shift in the maxima of the hyperfine field distributions with the increase of the tungsten content. This indicates similar magnetic properties of the amorphous phase for all alloy compositions.

**Figure 3:**
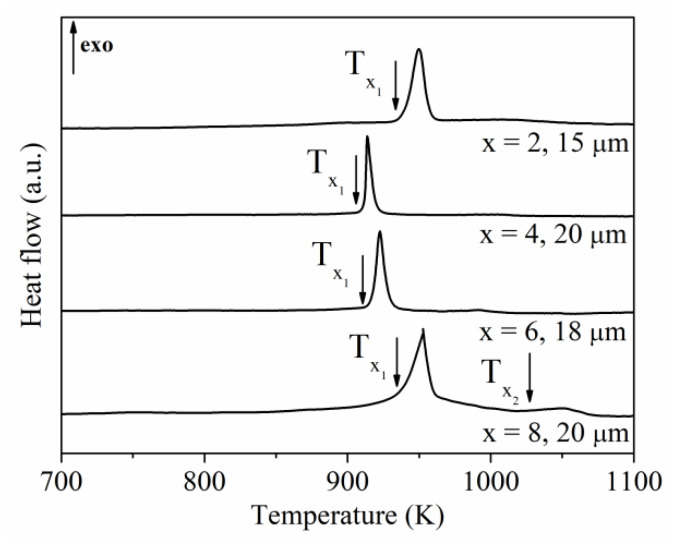
DSC curves of the Pr_9_Fe_65_W_x_B_26-x_ (where: x = 2, 4, 6, 8) alloy ribbons measured at a heating rate of 10 K/min ($T_{x_{1}}$ and $T_{x_{2}}$—first and second onset crystallization temperature).

**Figure 4:**
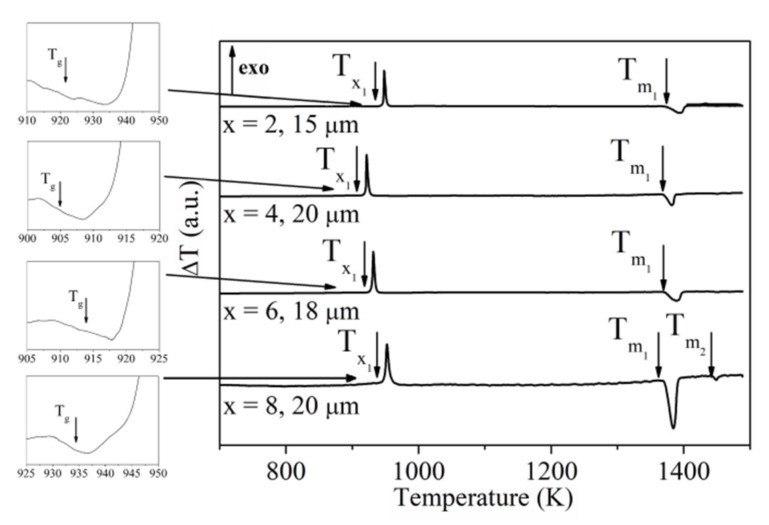
DTA curves of the Pr_9_Fe_65_W_x_B_26-x_ (where: x = 2, 4, 6, 8) alloy ribbons measured at a heating rate of 10 K/min (T_g_—glass transition temperature, $T_{x_{1}}$—first onset crystallization temperature, $T_{m_{1}}$ and $T_{m_{2}}$—first and second melting temperatures).

The DSC and DTA scans measured for the amorphous ribbon samples of all investigated compositions are presented in Figure 3 and Figure 4, respectively. These measurements allowed us to determine the glass transition temperature T_g_, onset crystallization temperature T_x_, melting temperature T_m_, supercooled liquid region ΔT_x_ = T_x_ − T_g_, reduced glass transition temperature T_rg_ = T_g_/T_m_ as well the glass forming ability parameter γ = T_x_/(T_g_ + T_m_) (Table 1) [41,42].

The changes of T_g_ and T_x_, as well as ΔT_x_, were observed with the change of W content. However, other parameters (T_m_, T_rg_ and γ) did not significantly change. The values of ΔT_x_ suggest that the Pr_9_Fe_65_W_x_B_26-x_ (where: x = 2, 4, 6, 8) alloys should not exhibit good glass forming abilities. On the other hand, high values of T_rg_ (~0.6) and γ (~0.3) indicate good glass forming abilities and a possibility of manufacturing amorphous samples with significant geometric dimensions [43,44,45]. However, our previous studies [22] of the Pr_9_Fe_65_W_x_B_26-x_ (where: x = 2, 4, 6, 8) alloys in the form of 1 mm rods and 0.5 mm thick plates produced using the suction-casting technique have shown partial crystallization of the as-cast specimens.

**Figure 5:**
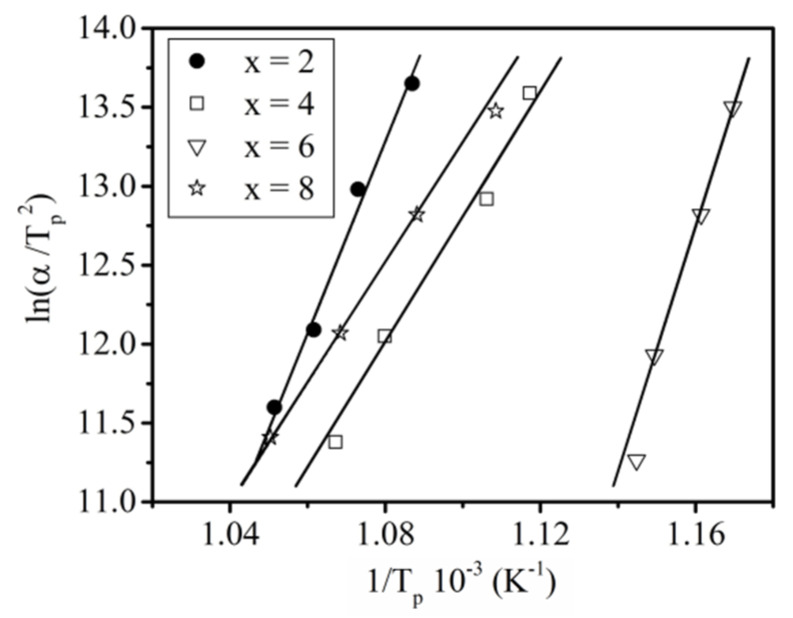
Kissinger plots for crystallization of the Pr_9_Fe_65_W_x_B_26-x_ (where: x = 2, 4, 6, 8) alloy ribbons (T_p_—the peak temperature corresponding to the maximum of the peaks on DSC curves).

The activation energies E_a_ for crystallization for each alloy compositions were calculated using the Kissinger analysis [46] base on the plots shown in Figure 5. The values of E_a_ were collected in Table 2. Error analysis was carried out using standard methods, taking into account apparatus input, apparatus calibration quality and subjective assessment of uncertainty associated with the determination of characteristic temperatures based on DSC curves. The results indicate that for Pr_9_Fe_65_W_4_B_22_ and Pr_9_Fe_65_W_8_B_18_ alloys, the crystallization processes feature lower values of E_a_ compared to those for the Pr_9_Fe_65_W_2_B_24_ and Pr_9_Fe_65_W_6_B_20_ alloys. These differences can be attributed to the crystallization of additional soft magnetic Fe_2_B or α-Fe phases during heat treatment of alloys containing 2 and 6 at. % of W, respectively (see later).

**Figure 6:**
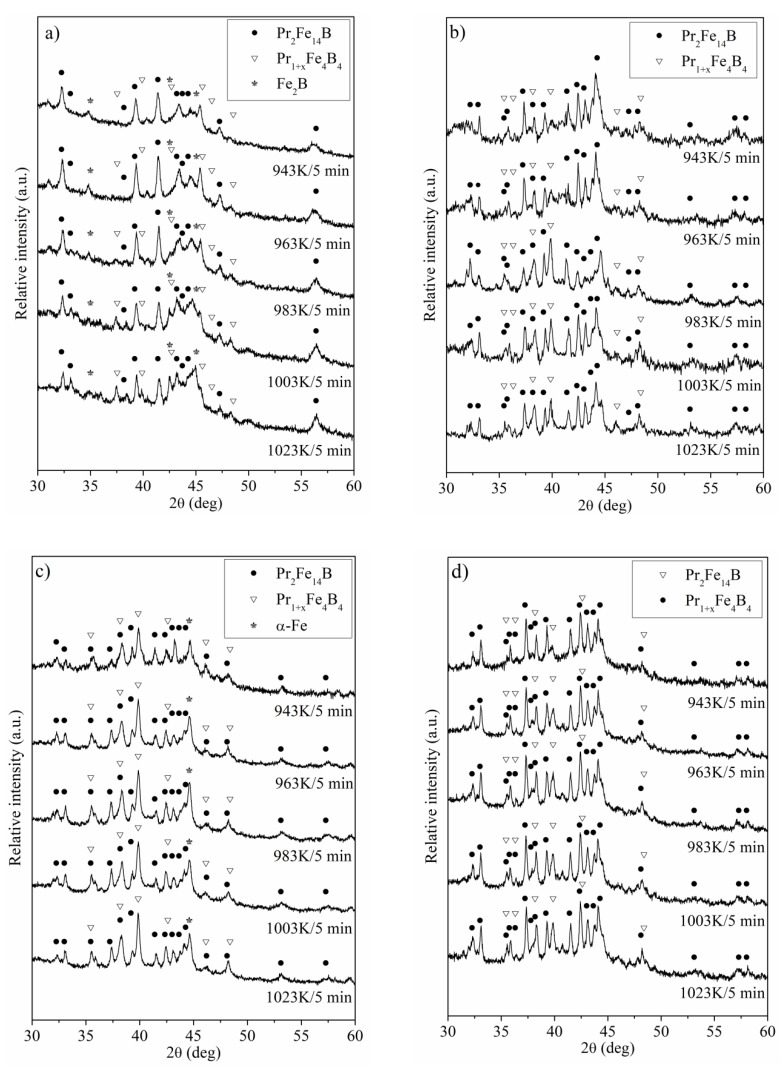
XRD patterns measured for the Pr_9_Fe_65_W_x_B_26-x_ (where: x = 2 (**a**), x = 4 (**b**), x = 6 (**c**) and x = 8 (**d**)) alloy ribbons subjected to annealing.

The XRD scans measured for Pr_9_Fe_65_W_x_B_26-x_ (where x = 2, 4, 6, 8) alloy ribbons subjected to annealing are shown in Figure 6. Short-time annealing of the specimens resulted in significant changes in their phase constitution. The crystalline phases detected for all annealed samples are the hard magnetic Pr_2_Fe_14_B and paramagnetic Pr_1+x_Fe_4_B_4_. Furthermore, the phase analysis for the Pr_9_Fe_65_W_2_B_24_ alloy ribbons has shown the presence of the reflexes corresponding to the soft magnetic Fe_2_B phase (Figure 6a), while for the Pr_9_Fe_65_W_6_B_20_ alloy, the α-Fe phase was detected (Figure 6d). Heat treatment at 943 K and higher temperatures resulted in nucleation and growth of the crystalline phases. The increase of annealing temperature caused changes in the volume fractions of the constituent phases, which was reflected in their magnetic properties. Moreover, for the Pr_9_Fe_65_W_2_B_24_ alloy, the widening of the diffraction peaks is related to low volume fractions and smaller grain sizes of the crystalline phases.

Based on the XRD studies of annealed Pr_9_Fe_65_W_x_B_26-x_ (where: x = 2, 4, 6, 8) ribbons, one can deduce that the wide crystallization peaks on the DSC and DTA curves corresponding to $T_{x_{1}}$ (Figure 3 and Figure 4) indicate the formation of the Pr_2_Fe_14_B and Pr_1+x_Fe_4_B_4_ phases. It is expected that the $T_{x_{2}}$ may be related to the decomposition of the hard magnetic Pr_2_Fe_14_B phase. This might be related to some oxidation processes leading to a reduction of the volume fraction of the hard magnetic phase. This might be a reason for the decrease of _J_H_c_ in ribbon annealed at 1023 K (see Table 4).

**Figure 7:**
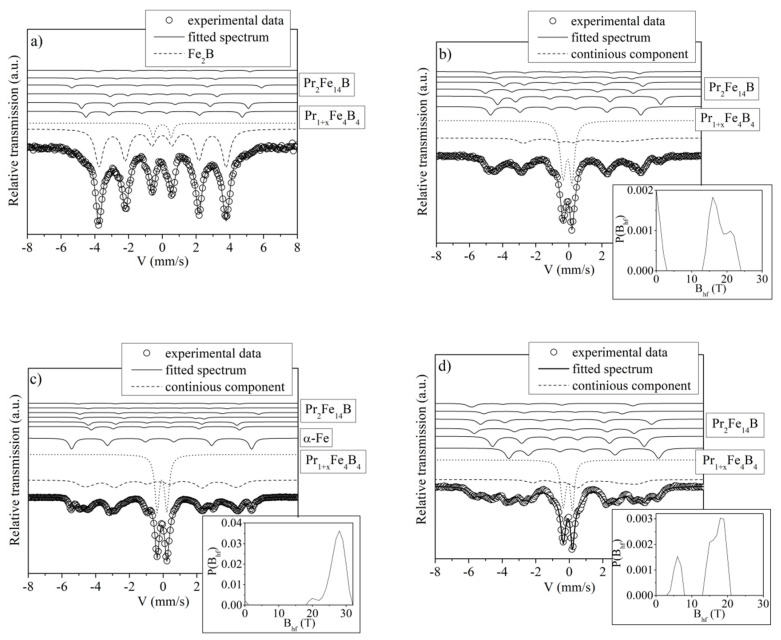
Mössbauer spectra with corresponding hyperfine field distributions of the Pr_9_Fe_65_W_x_B_26-x_ (where: x = 2 (**a**), x = 4 (**b**), x = 6 (**c**) and x = 8 (**d**)) ribbons annealed at 1003 K.

The transmission Mössbauer spectra measured for the Pr_9_Fe_65_W_x_B_26-x_ (where: x = 2, 4, 6, 8) ribbons annealed at 1003 K for 5 min are shown in Figure 7. The contribution from the hard magnetic Pr_2_Fe_14_B phase was represented by six sextets each of them corresponding to a distinct Fe site in this phase. They correspond to six nonequivalent positions of Fe atoms in its elementary cell. Base on the Wyckoff notation they are labeled as 16k_1_, 16k_2_, 8j_1_, 8j_2_, 4e and 4c, for which the relative intensity ratios of 4:4:2:2:1:1 were chosen. The standard fitting procedure for the Pr_2_Fe_14_B phase was based on assumptions introduced by Pinkerton et al. [47]. In order to match theoretical spectra to the experimental data, additional spectral components were added. Particularly, the doublet line corresponding to the paramagnetic Pr_1+x_Fe_4_B_4_ crystalline phase was appended to the fitting model [48]. In the case of the Pr_9_Fe_65_W_2_B_24_ alloy (Figure 7a), the presence of the soft magnetic Fe_2_B phase was also considered [49]. The Fe_2_B phase was represented by a single sextet. The quantitative analysis of the spectrum has shown that the Pr_9_Fe_65_W_2_B_24_ ribbon sample contains low volume fractions of the hard magnetic Pr_2_Fe_14_B (9 vol. %) and paramagnetic Pr_1+x_Fe_4_B_4_ (9 vol. %) phases. These calculations have also shown that the largest volume fraction corresponds to the soft magnetic Fe_2_B phase (82 vol. %) thus explaining relatively weak magnetic properties of the annealed Pr_9_Fe_65_W_2_B_24_ ribbons. The shape of the Mössbauer spectrum measured for the Pr_9_Fe_65_W_4_B_22_ alloy ribbon suggests the presence of a major fraction of crystalline phases (Figure 7b). In order to obtain a good fit an additional broad sextet line, defined by the hyperfine field distribution, was included. As there are limitations regarding the number of sub-spectra that can be independently fitted by the Mössbauer spectra analysis software, the parameters for the low-intensity sub-spectra corresponding to 4e and 4c positions of Fe atoms were arbitrary set. One could expect that the short-time heat treatment (for 5 min) led to the presence of a remnant amorphous phase. However, the broad sextet line more likely can be attributed to the highly disordered hard magnetic Pr_2_Fe_14_B phase, formed during short-time annealing and then rapid quenching in icy water. A narrow hyperfine field distribution (instead of a broad one, which is typical for the amorphous phase) was calculated. It can be an argument in favor of the formation of a highly disordered crystalline Pr_2_Fe_14_B phase. The Pr_9_Fe_65_W_4_B_22_ ribbon sample consists of 58 vol. % of the paramagnetic Pr_1+x_Fe_4_B_4_ phase, 36 vol. % of the hard magnetic Pr_2_Fe_14_B phase and 6 vol. % of the disordered phase. The Mössbauer spectrum of the Pr_9_Fe_65_W_6_B_20_ alloy ribbon (Figure 7c) has indicated the presence of the components corresponding to the hard magnetic Pr_2_Fe_14_B (13 vol. %), paramagnetic Pr_1+x_Fe_4_B_4_ (53 vol. %), highly disordered phase (23 vol. %) and a low volume fraction of soft magnetic α-Fe phase [50] (11 vol. %). The best fitting of the experimental data for the annealed ribbon of the Pr_9_Fe_65_W_8_B_18_ alloy (Figure 7d) was attained by taking into account three components—the paramagnetic Pr_1+x_Fe_4_B_4_, hard magnetic Pr_2_Fe_14_B and highly disordered phases. The corresponding volume fractions of the constituent phases were 37 vol. % of the paramagnetic Pr_1+x_Fe_4_B_4_ phase, 48 vol. % of the hard magnetic Pr_2_Fe_14_B phase and 15 vol. % of the highly disordered phase. A large volume fraction of the paramagnetic Pr_1+x_Fe_4_B_4_phase reduced the saturation magnetization J_s_. The presence of large volume fractions of hard magnetic and crystallographically highly disordered (also hard magnetic) phases were responsible for the ferromagnetic behavior of the investigated alloys. However, the volume fractions of the constituent phases changed significantly with the W addition, which was reflected in the magnetic properties of the investigated alloys.

The calculated hyperfine parameters (B_hf_—hyperfine field, IS—isomer shift, QS—quadrupole splitting) corresponding to the constituent phases of the Pr_9_Fe_65_W_x_B_26-x_ (where: x = 2, 4, 6, 8) ribbons annealed at 1003 K for 5 min are collected in Table 3. Maximum uncertainties for the calculated hyperfine parameters are ΔB_hf_ = ± 0.1 T for hyperfine field, ΔQS = ± 0.05 mm/s for the isomer shift, ΔIS = ± 0.002 mm/s for quadrupole splitting.

**Figure 8:**
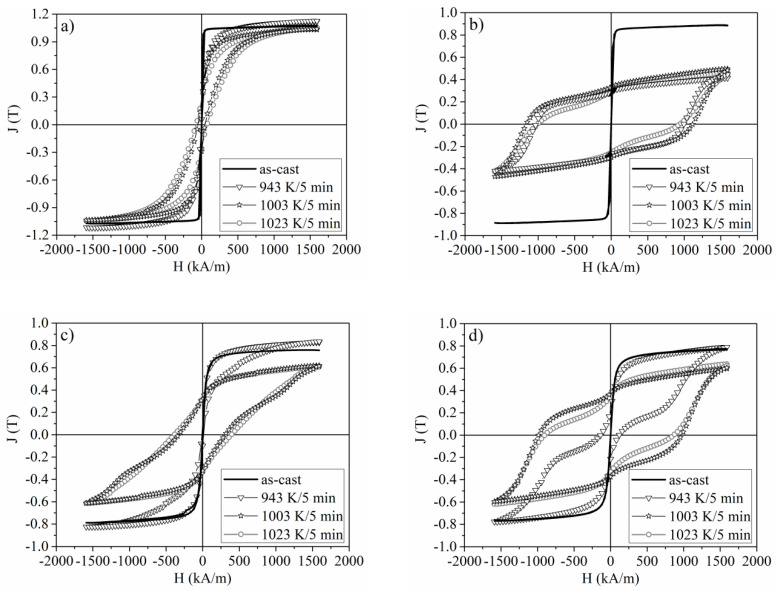
The selected hysteresis loops measured for the Pr_9_Fe_65_W_x_B_26-x_ (where: x = 2 (**a**), x = 4 (**b**), x = 6 (**c**) and x = 8 (**d**)) alloy ribbons.

The selected hysteresis loops J(H) measured for the Pr_9_Fe_65_W_x_B_26-x_ (where: x = 2, 4, 6, 8) alloy ribbons are shown in Figure 8. The hysteresis loops were measured as a dependence of magnetic polarization J vs. external magnetic field H, therefore, the determined coercive fields correspond to the magnetic field at which J reaches zero. J(H) measured for as-cast samples are characteristic of the soft magnetic materials. Annealing of ribbons containing 2 at. % of W did not cause significant changes in their magnetic properties. Low coercivity _J_H_c_ indicate a low volume fraction of a hard magnetic Pr_2_Fe_14_B phase, which was previously confirmed by the Mössbauer spectra analysis. A change of W content resulted in an evolution of microstructure and phase constitution of annealed specimens, thus leading to significant changes in their magnetic properties. Magnetic measurements carried out on ribbons containing 4, 6 and 8 at. % of W have shown their hard magnetic properties. Especially, an increase of the coercivity _J_H_c_ and maximum magnetic energy product (BH)_max_ with the increase of the annealing temperature was observed. However, for the x = 6 and x = 8 alloys annealed at 943 K, the wasp-waisted shapes of the hysteresis loops were measured. This suggests the formation of the hard magnetic Pr_2_Fe_14_B phase within the soft magnetic amorphous matrix. For the alloys containing 4 and 8 at. % of W annealed at higher temperatures, the presence of large volume fractions of the hard magnetic Pr_2_Fe_14_B phase resulted in wide hysteresis loops. Based on our Mössbauer studies (Table 3) one can assume that the improvement of _J_H_c_ is caused by the larger volume fraction of the hard magnetic phases in the vicinity of PrFe_4_B_4_ type borides in the x = 4 alloy, while the presence of α-Fe and Fe_2_B for the annealed samples of the x = 2 and x = 6 alloys, respectively, resulted in a higher J_r_ value. Higher (BH)_max_ for the x = 8 alloy might be somewhat related to a more homogeneous microstructure of the annealed specimens (see Figure 10). It was shown that the increase of W contents at the expense of Fe resulted in a decrease of the saturation polarization J_s_ of the as-cast specimens. The magnetic parameters measured for the Pr_9_Fe_65_W_x_B_26-x_ (where: x = 2, 4, 6, 8) alloy ribbons are collected in Table 4. Based on technical specifications of the LakeShore VSM 7307 magnetometer, uncertainties for the measurement of the magnetic field were taken as 1% of the readings from the apparatus. The maximum coercivity of _J_H_c_ = (1148 ± 12) kA/m was measured for the Pr_9_Fe_65_W_4_B_22_ alloy ribbon annealed at 1003 K, while the largest (BH)_max_ = (23 ± 3) kJ/m^3^ was determined for the Pr_9_Fe_65_W_8_B_18_ alloy ribbon annealed at 983 K.

**Figure 9:**
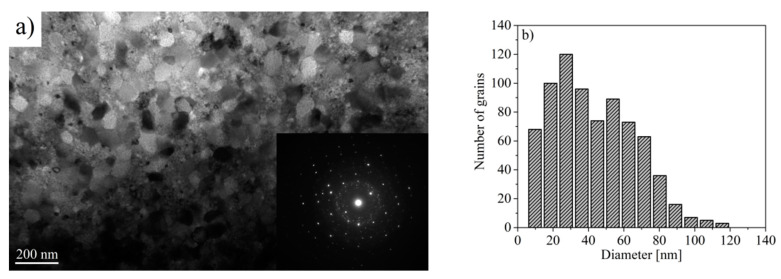
Transmission electron micrographs of the Pr_9_Fe_65_W_4_B_22_ alloy ribbon annealed at 1003 K together with the electron diffractograms (**a**) and the grain size distribution (**b**).

**Figure 10:**
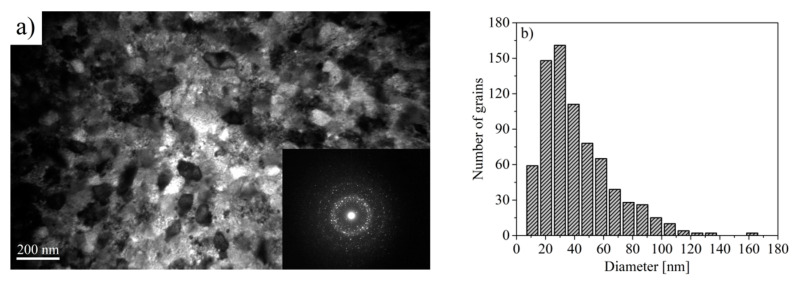
Transmission electron micrographs of the Pr_9_Fe_65_W_8_B_18_ alloy ribbon annealed at 1003 K together with the electron diffractograms (**a**) and the grain size distribution (**b**).

The microstructure of samples subjected to annealing was observed using a transmission electron microscope. A heterogeneous microstructure was observed for the Pr_9_Fe_65_W_4_B_22_ sample (Figure 9). Here, next to the large grains (diameter of ~70 nm), some small crystallites having diameters lower than 20 nm, were measured. Moreover, the distribution of grain sizes obtained for the Pr_9_Fe_65_W_4_B_22_ alloy ribbon differs from those obtained for the Pr_9_Fe_65_W_8_B_18_ alloy. For the Pr_9_Fe_65_W_8_B_18_ alloy, the TEM studies have revealed the existence of agglomerates having the same crystallographic orientation that can also impact the magnetic properties of the sample (Figure 10). In the case of the Pr_9_Fe_65_W_8_B_18_ alloy ribbon annealed at 1003 K, the grain size distribution was relatively wide with a maximum at ~30 nm (Figure 10b). It has to be pointed out that except for the phase composition the microstructure can also play a significant role in shaping the magnetic properties. The electron diffraction patterns obtained for the investigated samples confirmed the presence of crystalline phases and indicated their nanocrystalline structure. The presence of a diffused electron diffraction pattern for the x = 8 alloy was caused by a large fraction of crystallites with the sizes lower than 10 nm. In the case of the x = 4 alloy, the presence of crystallites larger than 80 nm may give an additional point diffraction pattern superimposed on the diffused diffraction pattern.

## 4. Conclusions

In the present study, the phase constitution, microstructure and magnetic properties of the nanocrystalline Pr_9_Fe_65_W_x_B_26-x_ (where: x = 2, 4, 6, 8) alloy ribbons in an as-cast state and subjected to annealing were investigated. XRD and Mössbauer spectroscopy have shown that the as-cast ribbons of all alloy compositions were fully amorphous. Moreover, the analysis of the Mössbauer spectra has shown the bimodal character of the hyperfine field distribution, suggesting the existence of nonequivalent surroundings of Fe atoms in the amorphous phase. The hysteresis loops measured for the as-cast samples were characteristic for soft magnetic materials. Heat treatment of the samples led to precipitation of various crystalline phases. The phase constitution changes with the composition of the alloys. However, for all annealed samples, the crystallization of the hard magnetic Pr_2_Fe_14_B and the paramagnetic Pr_1+x_Fe_4_B_4_ phases took place. Additionally, for Pr_9_Fe_65_W_2_B_24_ and Pr_9_Fe_65_W_6_B_20_ alloy ribbons, the soft magnetic Fe_2_B and the α-Fe phases were observed. The crystallization of these additional crystalline phases during heat treatment can be related to higher values of the activation energy E_a_ for crystallization. An increase in tungsten content, as well as the rise of annealing temperature, resulted in the improvement of the magnetic properties. The maximum value of coercivity _J_H_c_ = (1148 ± 12) kA/m was measured for the Pr_9_Fe_65_W_4_B_22_ alloy ribbon annealed at 1003 K, whereas the largest maximum magnetic energy product (BH)_max_ = (23 ± 3) kJ/m^3^ was determined for the Pr_9_Fe_65_W_8_B_18_ alloy ribbon annealed at 983 K. The TEM studies revealed a heterogeneous mixture consisting of large (of diameter of ~70 nm) and small (of the diameter < 20 nm) crystallites for the Pr_9_Fe_65_W_4_B_22_ alloy. For the Pr_9_Fe_65_W_8_B_18_ ribbon, the largest number of grain reached sizes of ~30 nm. TEM dark-field studies of the Pr_9_Fe_65_W_8_B_18_ alloy specimen revealed a presence of agglomerates having the same crystallographic orientation. This might be a reason for the highest (BH)_max_ measured for the ribbon annealed at 1003 K.

## Figures and Tables

**Table 1:** Thermal stability parameters determined for amorphous ribbons of Pr_9_Fe_65_W_x_B_26-x_ (where: x = 2, 4, 6, 8) alloys: T_g_—glass transition temperature, $T_{x_{1}}$ and $T_{x_{2}}$—first and second onset crystallization temperature, $T_{m_{1}}$ and $T_{m_{2}}$—first and second melting temperatures, ΔT_x_ = T_x_ − T_g_ —supercooled liquid region, T_rg_ = T_g_/T_m_—reduced glass transition temperature, γ = T_x_/(T_g_+T_m_)—glass forming ability parameter.

x (at. %)	T_g_ (K)	$T_{x_{1}}(K)$	$T_{x_{2}}(K)$	T_m_ (K)	ΔT_x_ (K)	T_rg_	γ
2	920	940	-	1371	20	0.67	0.41
4	905	915	-	1371	10	0.66	0.40
6	914	920	-	1369	6	0.67	0.40
8	934	945	1020	1370	11	0.68	0.41

**Table 2:** The activation energies E_a_ for crystallization for the Pr_9_Fe_65_W_x_B_26-x_ (where: x = 2, 4, 6, 8) alloy ribbons.

x (at. %)	E_a_ (kJ/mol)
2	544 ± 11
4	330 ± 7
6	644 ± 13
8	320 ± 7

**Table 3:** The hyperfine parameters of the Pr_9_Fe_65_W_x_B_26-x_ (where: x = 2, 4, 6, 8) alloy ribbons annealed at 1003 K (B_hf_—hyperfine field, IS—isomer shift, QS—quadrupole splitting, V—fractions of constituent phase).

x	Identified Phases		B_hf_ (T)	IS (mm/s)	QS (mm/s)	V (vol. %)
2	Pr_2_Fe_14_B	16k_1_	28.7	−0.19	0.58	9
		16k_2_	30.6	0.07	0.18	
		8j_1_	19.6	−0.07	0.29	
		8j_2_	34.8	−0.17	0.85	
		4e	25.6	−0.64	−0.67	
		4c	27.9	0.80	−0.21	
	Pr_1+x_Fe_4_B_4_		-	−0.01	1.08	9
	Fe_2_B		23.3	−0.00	0.03	82
4	Pr_2_Fe_14_B	16k_1_	28.1	−0.25	0.13	36
		16k_2_	30.6	0.13	0.99	
		8j_1_	27.7	−0.71	0.28	
		8j_2_	26.0	0.53	0.01	
		4e	25.6	0.01	−0.67	
		4c	28.1	−0.15	−0.21	
	Pr_1+x_Fe_4_B_4_		-	−0.07	0.53	58
	disordered Pr_2_Fe_14_B		-	-	-	6
6	Pr_2_Fe_14_B	16k_1_	27.2	−0.14	0.55	13
		16k_2_	27.3	−0.14	0.23	
		8j_1_	22.7	−0.71	−1.05	
		8j_2_	32.9	0.40	−0.09	
		4e	25.6	0.01	−0.67	
		4c	29.6	−0.15	−0.21	
	Pr_1+x_Fe_4_B_4_		-	−0.07	0.58	53
	α-Fe		33.1	−0.15	0.12	11
	disordered Pr_2_Fe_14_B		-	-	-	23
8	Pr_2_Fe_14_B	16k_1_	27.7	0.50	0.71	48
		16k_2_	28.0	−0.14	0.17	
		8j_1_	29.6	−0.70	−0.40	
		8j_2_	31.6	−0.54	0.69	
		4e	25.6	−0.63	−0.67	
		4c	29.6	−2.09	2.22	
	Pr_1+x_Fe_4_B_4_		-	−0.08	0.55	37
	disordered Pr_2_Fe_14_B		-	-	-	15

**Table 4:** Magnetic parameters: coercivity _J_H_c_, remanence polarization J_r_, maximum magnetic energy product (BH)_max_ and saturation polarization J_s_ for annealed ribbon samples.

x (at. %)	T (K)	_J_H_c_ (kA/m)	J_r_ (T)	(BH)_max_ (kJ/m^3^)	J_s_ (T)
2	943	3	0.05	-	1.12
	963	3	0.07	-	1.12
	983	21	0.17	-	1.13
	1003	60	0.35	3	1.04
	1023	77	0.33	4	1.04
4	943	1013	0.29	14	0.42
	963	1053	0.30	16	0.45
	983	975	0.29	14	0.48
	1003	1148	0.30	13	0.49
	1023	972	0.30	13	0.45
6	943	14	0.01	-	0.83
	963	204	0.26	6	0.58
	983	390	0.33	1	0.61
	1003	319	0.32	11	0.62
	1023	359	0.32	10	0.66
8	943	136	0.25	2	0.77
	963	910	0.40	22	0.63
	983	960	0.39	23	0.62
	1003	983	0.37	20	0.60
	1023	882	0.34	15	0.63

## References

[1] Herbst J.F., Croat J.J. (1991). Neodymium-iron-boron permanent magnets. J. Magn. Magn. Mater..

[2] Sagawa M., Fujimura S., Togawa M., Yamamoto H., Matsuura Y. (1984). New material for permanent magnets on a base of Nd and Fe. J. Appl. Phys..

[3] Croat J.J., Herbst J.F., Lee R.W., Pinkerton F.E. (1984). Pr-Fe and Nd-Fe based materials: A new class of high- performance permanent magnets. J. Appl. Phys..

[4] Manaf A., Buckley R.A., Davies H.A., Leonowicz M. (1991). Enhanced magnetic properties in rapidly solidified Nd-Fe-B based alloys. J. Magn. Magn. Mater..

[5] Betancourt J.I., Davies H.A. (1999). Magnetic properties of nanocrystalline didymium (Nd–Pr)–Fe–B alloys. J. Appl. Phys..

[6] Stadelmaier H.H., Henig E.T., Petzow G. (1991). A Chronicle of the development of iron based rare earth high-performance magnets. Z. Metallkd..

[7] Herbst J.H., Yelon W.B. (1985). Crystal and magnetic structure of Pr_2_Fe_14_B and Dy_2_Fe_14_B. J. Appl. Phys..

[8] Wohlfarth E.P., Buschow K.H.J. (1988). Handbook of Magnetic Materials.

[9] Hirosawa S., Matsuura Y., Yamamoto H. (1986). Magnetization and magnetic anisotropy of R_2_Fe_14_B measured on single crystals. J. Appl. Phys..

[10] Liu Z., Davies H.A. (2004). Composition and microstructure dependent spin reorientation in nanocrystalline (Nd-Pr)-(Fe-Co)-B alloys. IEEE Trans. Magn..

[11] Mendoza-Suárez G., Davies H.A., Escalante-García J.I. (2000). _J_Hc, J_r_and (BH)_max_ relationship in PrFeB melt spun alloys. J. Magn. Magn. Mater..

[12] You C., Tian N., Lu Z., Ge L., Jing X. (2010). B effects on crystallization and magnetic properties of Pr_9.44_Fe_90.56-x_B_x_ (x = 7.16, 4.76) nanocomposite magnets. Acta Metall. Sin..

[13] Wang Z.C., Davies H.A. (2004). Structural and magnetic property evolution of Pr-Fe-Co-B hard magnetic alloys during devitrification annealing. Mater. Sci. Eng. A.

[14] Chang H.W., Chiu C.H., Chang C.W., Chen C.H., Chang W.C., Yao Y.D., Sun A.C. (2006). Effect of substitution of refractory elements for Fe on the magnetic properties of melt-spun Pr_9.5_Fe_80.5_B_10_ nanocomposites. J. Alloys Compd..

[15] Ren W.J., Zhang Z.D., Zhao X.G., You C.Y., Geng D.Y. (2003). Structure, magnetic properties and magnetostriction of Dy_1-x_Pr_x_(Fe_0.9_B_0.1_)_1.93_ (0 ≤ x ≤ 0.8) alloys. J. Alloys Compd..

[16] Chang H.W., Shih M.F., Chang C.W., Hsieh C.C., Cheng Y.T., Chang W.C., Sun A.C., Yao Y.D. (2008). Magnetic properties, phase evolution and microstructure of directly quenched bulk Pr-Fe-B-Nb magnets. Scr. Mater..

[17] Gabay A.M., Zhang Y., Hadjipanayis G.C. (2006). Effect of Cu and Ga addition on the anisotropy of R_2_Fe_14_B/α-Fe nanocomposite die-upset magnets (R = Pr, Nd). J. Magn. Magn. Mater..

[18] Yang B., Shen B.G., Zhao T.Y., Sun J.R. (2007). Significant improvement of structure and magnetic properties of Pr_2_Fe_14_B/α-Fe nanocomposite magnets due to Cu and Mn substitution. Mater. Sci. Eng. B.

[19] Przybył A., Wysłocki J.J., Kaszuwara W., Leonowicz M. (2005). Influence of tungsten content on magnetization processes in nanocomposite Nd_2_Fe_14_B/α-Fe magnets. Arch. Mater. Sci..

[20] Girt E., Altounian Z., Ryan D.H. (1998). Fe-substitution in Nd_2_Fe_17-__δ_X_δ_C_0.3_ (X = Al, Ti, V, W and δ = 0, 0.5). J. Magn. Magn. Mater..

[21] Yan A., Song X., Wang X. (2000). Segregation of W in Nd-Fe-B magnets and its effect on coercivity. IEEE Trans. Magn..

[22] Filipecka K., Pawlik P., Pawlik K., Gebara P., Przybył A., Pruba M. (2015). Effect of tungsten addition on phase constitution and magnetic properties of the bulk Fe_65_Pr_9_B_26-x_W_x_ alloys. Acta Phys. Pol. A.

[23] Filipecka K., Pawlik P., Kozdraś A., Filipecki J., Pawlik K., Wysłocki J.J. (2017). Magnetic properties and phase constitution of the nanocrystalline Fe_65_Pr_9_B_18_W_8_ alloy ribbons. Acta Phys. Pol. A.

[24] Filipecka K., Pawlik P., Filipecki J. (2017). The effect of annealing on magnetic properties, phase structure and evolution of free volumes in Pr-Fe-B-W metallic glasses. J. Alloys Compd..

[25] Filipecka K., Pawlik P., Kaszuwara W., Kozdraś A., Pawlik K., Filipecki J. (2019). Magnetic and structural studies of the rapidly solidified amorphous and nanocrystalline Pr_9_Fe_65_W_4_B_22_ alloys. J. Non-Cryst. Solids.

[26] Pawlik P. (2006). Soft magnetic Fe–Co–Zr–W–B bulk glassy alloys. J. Alloys Compd..

[27] Przybył A., Wysłocki J.J. (2006). The effect of annealing temperature on structure and magnetic properties of nanocomposite Nd_10_Fe_84−x_W_x_B_6_ (0 < x < 33 at. %) magnets. J. Mater. Process. Technol..

[28] Przybył A., Wnuk I., Gębara P., Wysłocki J.J. (2011). Effect of grain size and tungsten addition on microstructure and magnetic properties of Nd-FeB type magnets. J. Achiev. Mater. Manuf. Eng..

[29] Pawlik P., Pawlik K., Davies H.A., Kaszuwara W., Wysłocki J.J., Harrison N., Todd I. (2006). Directly quenched bulk nanocrystalline (Pr, Dy)–(Fe, Co)–B–Zr–Ti hard magnets. J. Alloys Comp..

[30] Yao B., Si L., Tan H., Zhang Y., Li Y. (2003). Effects of high boron content on crystallization, forming ability and magnetic properties of amorphous Fe_91-x_Zr_5_B_x_Nb_4_ alloy. J. Non-Cryst. Solids.

[31] Zhang W., Inoue A. (2002). Bulk nanocomposite permanent magnets produced by crystallization of (Fe,Co)–(Nd,Dy)–B bulk glassy alloy. Appl. Phys. Lett..

[32] Ishihara S., Zhang W., Inoue A. (2002). Hot pressing of Fe–Co–Nd–Dy–B glassy powders in supercooled liquid state and hard magnetic properties of the consolidated alloys. Scr. Mater..

[33] Pawlik P., Pawlik K., Davies H.A., Kaszuwara W., Wysłocki J.J. (2007). The influence of heat treatment on the microstructure and magnetic properties of (Fe,Co)–Zr–(Pr,Dy)–B- nanocomposite alloys. J. Magn. Magn. Mater..

[34] Inoue A., Takeuchi A., Makino A., Masumoto T. (1995). Hard magnetic properties of Fe-Nd-B alloys containing intergranular amorphous phase. IEEE Trans. Magn..

[35] Manaf A., Buckley R.A., Davies H.A. (1993). New nanocrystalline high-remanence Nd-Fe-B alloys by rapid solidification. J. Magn. Magn. Mater..

[36] Zhang W., Matsushita M. (2001). Hard magnetic properties of Fe–Co–Nd–Dy–B nanocrystalline alloys containing residual amorphous phase. J. Appl. Phys..

[37] Thamm S., Hesse J. (1996). A simple plot indicating interactions between single domain particles. J. Magn. Magn. Mater..

[38] Chrobak A., Ziółkowski G., Randrianantoandro N., Klimontko J., Chrobak D., Prusik K., Rak J. (2015). Ultra-high coercivity of (Fe_86−x_Nb_x_B_14_)_0.88_Tb_0.12_ bulk nanocrystalline magnets. Acta Mater..

[39] Pawlik K., Pawlik P., Wysłocki J.J., Kaszuwara W. (2020). Structural and magnetic studies of bulk nanocomposite magnets derived from rapidly solidified Pr-(Fe,Co)-(Zr,Nb)-B alloy. Materials.

[40] Filipecka K., Pawlik K., Pawlik P., Wysłocki J.J., Gębara P., Przybył A. (2014). Phase composition and magnetic properties of the nanocrystalline Fe_64,32_Pr_9,6_B_22,08_W_4_ alloy. Acta Phys. Pol. A.

[41] Lu Z.P., Liu C.T. (2004). A new approach to understanding and measuring glass formation in bulk amorphous materials. Intermetallics.

[42] Kao S.W., Yang K.C., Wang S.H., Hwang C.C., Lee P.Y., Huang R.T., Chin T.S. (2009). Predicting the glass-forming-ability of alloys by molecular dynamics simulation: A working example of Ti–Co bulk metallic glasses. Jpn. J. Appl. Phys..

[43] Turnbull D., Fisher J.C. (1949). Rate of nucleation in condensed systems. J. Chem. Phys..

[44] Davies H.A. (1976). Formation of metallic glasses. Phys. Chem. Glasses.

[45] Lu Z.P., Liu C.T., Thompson J., Porter W.D. (2004). Structural amorphous steels. Phys. Rev. Lett..

[46] Kissinger H.E. (1957). Reaction kinetics in differential thermal analysis. Anal. Chem..

[47] Pinkerton F.E., Dunham W.R. (1985). Mössbauer effect in R_2_Fe_14_B compounds. J. Appl. Phys..

[48] Saccone F.D., Torres C.E.R., Sanchez F.H., Gutfleisch O. (2002). Observation of hydrogen induced intermediate borides in PrFeB based alloys by Mossbauer effect spectroscopy. Physica B.

[49] Budzyński M., Constantin V.C., Popescu A.M.J., Surowiec Z., Tkachenka T.M., Yanushkevich K.I. (2015). Mössbauer study of treated Nd_2_Fe_14_B. Nukleonika.

[50] Jartych E., Kubalova L.M., Fadeeva V.I. (2015). X-ray diffraction and Mössbauer spectroscopy studies of mechanosynthesized Fe_75_B_25_ alloy. Nukleonika.

